# Supplementary material for: Factors influencing fans’ spectating experience and configuration effects in CBA league
Source: PLoS One. 2025 Jan 3;20(1):e0316706. doi: 10.1371/journal.pone.0316706 (PMC11698371; doi:10.1371/journal.pone.0316706)
Supplement: S1 Appendix — (DOCX) [file pone.0316706.s001.docx]

**Questionnaire on CBA League Fans' Satisfaction with On-site spectating Experience**

Dear Fan Respondents:

This is an anonymous questionnaire for CBA League fans' satisfaction with their on-site viewing experience. The purpose of this survey is to better understand the factors influencing the on-site viewing experience of CBA League fans. Before filling out the questionnaire, you can fully read the informed consent form and the questionnaire content, and choose whether to participate in the survey voluntarily. There is no right or wrong answer in this questionnaire, just answer according to your actual feeling of watching the game on the spot. Your answers have important reference value for this study, we look forward to your true expression of your thoughts and feelings, thank you for your support and help!

When choosing answers, 1, 2, 3, 4 and 5 represent strongly disagree, disagree, generally, agree, strongly agree and absolutely impossible, impossible, generally, possible, very likely respectively.

**Informed consent form**

Research Study：Factors influencing fans’ spectating experience and configuration effects in CBA league

Name of researcher：Duan Yu、Hongwei Fan、Ning Zhang

**Please read the informed consent form carefully and choose whether or not to participate voluntarily！**

| I am volunteering to participate in this questionnaire. | YES/NO |
| --- | --- |
| I understand that I am free to decline to participate in this research study, or I may withdraw my participation at any point without penalty. | YES/NO |
| I am aware of, and have been fully informed by the researcher of, the personal information to be obtained for this study, including gender, age, and education. | YES/NO |
| I have fully read the contents of this questionnaire and have chosen to voluntarily participate in this survey. | YES/NO |
| I authorize and consent to the researcher's use of my completed questionnaire for academic research purposes. | YES/NO |

| Variables | Item |
| --- | --- |
| SENSE | S1: I think CBA league arenas are clean and well-designed |
|  | S2: The lighting effects in the CBA league arena immersed me in it |
|  | S3: The acoustics in CBA league arenas make me feel uplifted |
|  | S4: I feel enriched by the information provided by the scoreboards in CBA league arenas |
|  | S5: The live atmosphere of the CBA league makes me feel great |
|  | S6: The live hosts of the CBA league were able to lead me into the atmosphere of the game and made me feel excited |
| FEEL | F1: I'll get excited or depressed about a particular emotion as the game goes on |
|  | F2: The staff at CBA league venues has a high level of service and made me feel friendly and approachable |
|  | F3: After watching the game live, I will feel happy or happy both physically and mentally |
|  | F4: I get bored and don't have any mood swings when watching the game live |
|  | F5: I'm proud of my favorite team when they have a better record |
| THINK | T1: Before watching the game live, I would be curious about the outcome of the game and the performance of the players |
|  | T2: While watching the game, I will be thinking about how the team can get the win |
|  | T3: After watching the game live, I will think about the reasons why my team lost the game |
|  | T4: After watching the game live, it will give me a better understanding of the rules of basketball competition as well as the rules of officiating |
| ACT | A1: After watching the game live, I will take the initiative to share the experience and feelings of watching the game with others |
|  | A2: The slogan and the spirit of the CBA league will change my attitude towards life |
|  | A3: After watching the game live, I would want to start learning basketball skills |
|  | A4: When things get tough in life, I motivate myself with the players' hard work and their spirit of not giving up |
| RELATE | R1: After watching the game, I will want to buy souvenirs or derivatives related to the CBA league. |
|  | R2: I will actively cheer for my favorite player or team during the live game. |
|  | R3: I will develop a liking for other fans who have had the experience of attending the same game and will establish interactive behaviors |
|  | R4: Watching the game live will give me a sense of belonging to a group with other fans |
| Spectator Service | SS1: The security staff at the CBA league venues were able to maintain order on the floor better and made me feel safe |
|  | SS2: The accessibility of CBA league venues and the ease of getting in and out of the venues is better |
|  | SS3: Before the game, the arena staff was able to guide me quickly to my seat and watch the game |
|  | SS4: CBA league venues are able to offer a wide variety and better quality of food and beverages |
|  | SS5: I was able to quickly purchase tickets and get information about the event before attending the game |
| Spectator Experience | SE1: What is the likelihood that you would buy tickets again to watch a CBA event live? |
|  | SE2: The likelihood that you'll be going to live games regularly in the future? |
|  | SE3: What is the likelihood that you would recommend others to go to a live CBA event? |
|  | SE4: For the most part, the live viewing experience in the CBA league is satisfactory to me |
