# Supplementary material for: Factors influencing fans’ spectating experience and configuration effects in CBA league
Source: PLoS One. 2025 Jan 3;20(1):e0316706. doi: 10.1371/journal.pone.0316706 (PMC11698371; doi:10.1371/journal.pone.0316706)
Supplement: Attachment — Submitted filename: Response to Reviewers.docx [file pone.0316706.s003.docx]

Response letter

Dear Editor Vincenzo Auriemma and Reviewers:

On behalf of my co-authors, we sincerely appreciate you and the reviewers for your precious time in reviewing our paper and giving us another chance to revise our manuscript. It was your valuable and insightful comments that led to possible improvements in the revised paper. According to you and the reviewers’ comments, we have carefully considered the comments and tried our best to address every one of them. We hope the manuscript after careful revisions can meet your high standards and expectations. Below we provide the point-by-point responses, all modifications in the manuscript have been highlighted in yellow.

Once again, we would like to express our great appreciation to you and the reviewers for your warm work and comments on our paper. We look forward to hearing from you!

Thank you and best regards,

Ning Zhang, Ph.D.

Corresponding author:

Name: Ning Zhang

E-mail: [417997356@qq.com](mailto:417997356@qq.com)

**Response to Editor**

***Comment #1. Please ensure that your manuscript meets PLOS ONE's style requirements, including those for file naming.***

**Response:** Thanks for your kind reminders. We had adjusted the manuscript’s style according to journal requirements, including manuscript body formatting and title, author and affiliations formatting. We’re welcome for any adjustment if still needed.

***Comment #2. Please provide additional details regarding participant consent. In the ethics statement in the Methods and online submission information, please ensure that you have specified (a) whether consent was informed and (b) what type you obtained (for instance, written or verbal, and if verbal, how it was documented and witnessed). If your study included minors, state whether you obtained consent from parents or guardians. If the need for consent was waived by the ethics committee, please include this information.***

**Response:** Thank you very much for your kind reminder and valuable comment! According to your comment, we had provided additional details about participant consent, including the type of informed consent and informed consent of minor participants. The revised content can be found in 4.1 Ethics statement.

**[Pg16, Ln5-8]: “**Written consent was obtained from the participants prior to the start of the study, and all participants signed a study-related informed consent form and chose to voluntarily participate in the questionnaire. If the participants were aged <18 years, consent was obtained from the participants and their guardians.**”**

***Comment #3.  Please include your full ethics statement in the ‘Methods’ section of your manuscript file. In your statement, please include the full name of the IRB or ethics committee who approved or waived your study, as well as whether or not you obtained informed written or verbal consent. If consent was waived for your study, please include this information in your statement as well.***

**Response:** Thank you very much for your precious comments! According to your comments, the full ethics statement and the full name of the ethics committee has been added to the methods section of our manuscript file. and state that written consent for this study was obtained from all participants. The full ethics statement as follows:

**[Pg16, Ln3-10]: “**The study was conducted with the approval of the Ethics Review Committee of the Chengdu Sport University (No. 2024106) to which the principal investigator belongs. Written consent was obtained from the participants prior to the start of the study, and all participants signed a study-related informed consent form and chose to voluntarily participate in the questionnaire. If the participants were aged <18 years, consent was obtained from the participants and their guardians. In addition, a certificate of exemption from ethical review issued by the Ethical Review Committee of Chengdu Sport university was obtained for this study.**”**

**Response to Reviewer#1**

Dear Reviewer:

We sincerely appreciate for your endorsement of our manuscript! Thank you for your professional work and dedication during the review process! It is your constructive and valuable comments that help us to improve our manuscript, and we sincerely hope our revised manuscript can meet your expectations and satisfaction!

***Comment #1. The article titled Factors influencing fans' spectating experience and configuration effects in CBA League is interesting. I do not know the length for publication. This article appears really long, with over 50 pages.***

**Response：**We sincerely appreciate your valuable time in reviewing our manuscript! And we completely understand your concerns. However, each data analysis step could not be dispensed with in order to ensure the rigor of the data analysis as well as the robustness of the study's conclusions, thus resulting in a longer article. On this basis, we still try our best to reduce the length of some parts of the article. We hope that our solution will be understood and supported by you! and furthermore, according to journal requirements, the word count and length of the manuscript have no restrictions.

***Comment #2. Use of abbreviations: Some abbreviations, like CBA (Chinese Basketball Association), are used inconsistently with the full term, even after being introduced. For example, "CBA league" is mentioned in various forms (CBA, Chinese Basketball Association, etc.), which could be unified for better readability.***

**Response:** Thank you very much for your precious comment and kind reminder! According to your comments, we had unified the abbreviation of CBA League in our manuscript for better readability. The full term of CBA League (China Basketball Association) was introduced in introduction and other mentioned of China basketball Association has been unified in CBA League. Besides, the abbreviation of research method also unified in Structural Equation Modeling (SEM) and Fuzzy-set Qualitative Comparative Analysis (fsQCA). We sincerely hope that the solution to this problem can meet your satisfaction, and we are welcome for any needed suggestions!

***Comment #3.  I would suggest to rewrite this sentence 'The findings of this study provide theoretical guidance and ideas for the enhancement of fans' spectator experience, and realize the practical needs of cultivating loyal fan groups of CBA league and expanding the influence of CBA itself.' as '******The findings provide theoretical guidance for enhancing fan experience and meeting the practical needs of cultivating loyal CBA fan groups while expanding the league's influence.'***

**Response:** Thank you for your valuable comments. Your insightful comment did help us to improve the quality of the paper. According to your comments, we rewrite the original sentence as “The findings provide theoretical guidance for enhancing fan experience and meeting the practical needs of cultivating loyal CBA fan groups while expanding the league's influence.” The revised sentence in the manuscript is as follows:

**[Pg2, Ln2-4]: “**The findings provide theoretical guidance for enhancing fan experience and meeting the practical needs of cultivating loyal CBA fan groups while expanding the league's influence.**”**

***Comment #4.  The sentence 'The analysis of necessity conditions shows that individual influencing factors do not constitute the necessary conditions for high satisfaction of Fans’ Spectator Experience.' and 'Starting from the group perspective, we use the empirical analysis method combining SEM and fsQCA to clarify the complex causal relationship' are not clear. I would recommend the authors to rewrite these two sentences.***

**Response:** Thank you very much for your valuable comments. It is Your insightful comments that led to the possible improvement of our revised manuscript. According to your comments, we had rewritten these two sentences as follows:

**Sentence #1 [Pg1, Ln19-22]: “**Among six influencing factors, none of them is the necessary condition for high satisfaction of Fans’ Spectator Experience. In this way, the characteristics of multi-factors and different paths of the live Spectator experience were verified.**”**

**Sentence #2 [Pg1, Ln13-15]: “**Using analysis method SEM and fsQCA, this study clarifies the complex causal relationship behind the phenomenon of fans' spectator experience, explore the linkage mechanism between different influencing factors of spectator experience.**”**

***Comment #5. The questionnaires results are not always reliable; the study would be stronger with a more diverse data collection with a triangulation methodology, such as interviews, observations, or cross-validation with ticket sales or fan engagement metrics. If the authors can not do this in this article, I would recommend the authors to put this as one of the limitation of this research.***

**Response:** Thank you very much for your insightful comments! Your suggestions are definitely feasible to enhance the quality of our revised manuscript. Regrettably, constraints have prevented us from resuming a more diverse collection of data. In future studies, we will adopt the diverse data collection that you suggest, then to improve the effectiveness of our study. But in order to verify the reliability of the questionnaire results, we conducted a common method bias test. At last, we had put your recommendation as one of the limitations in our manuscript, we sincerely hope our revised manuscript can meet your satisfaction!

**[Pg36, Ln3-7]: “**This study has the following limitations: (1) Although this study conducted a common method bias test for the questionnaire results, the effectiveness of the study could have been improved by using more diverse methods of data collection and triangulation, such as interviews, observations, and cross-validation of different indicators.**”**

***Comment #6. I would also suggest the authors consider other external factors like broader economic conditions, public interest in basketball, and global sports trends are not considered in the study. These external influences might play a significant role in shaping fan experiences, but the research focuses only on internal factors related to the games and services.***

**Response:** Thank you very much for your precious and insightful comments, it is your comments that lead to the possible improvement to the quality of our revised manuscript! According to your comments, we have discussed the external influences related to fans' live viewing experience in the discussion section, including the influence of external factors such as sporting event prestige, sporting experience and regional cultural characteristics on the live viewing experience. In future research, we will incorporate your suggestions to include more external influences in our empirical data analyses so that we can produce a study with broader applicability, In addition, we also include this as one of the limitations of this study.

**[Pg34, Ln10-19]: “**In addition to internal influences such as game and service, some external factors may also affect the CBA league fans' on-site viewing experience. For example, perceptions of event prestige play an important role in influencing fans’ willingness to continue to attend games, and sports event organizers can adopt different strategies to develop events and maintain fans’ interest in basketball [61]. Sport experience and spectator type, on the other hand, mediate the emergence of event nostalgia, as spectators with the same sport experience tend to be more loyal to the event [62]. Additionally, regional cultural identities also influence the sensory experience of the fan base, and the effectiveness of the sensory-centered experience is enhanced when regional cultural identities and elements are integrated into the arena layout [63].**”**

**[Pg36, Ln7-10]: “**Due to the operationalization of variables and the difficulty of data collection, relevant external influences were not included in this study. Future studies may consider incorporating external factors such as regional economic development, public interest in basketball, and global sports development patterns.**”**

**Response to Reviewer #2**

Dear Reviewer:

Thank you for your timely follow-up during the review process of this paper, we sincerely appreciate for your dedicated and valuable time! It is your valuable and precious comments that helped greatly in improving our manuscript. After studying your comments carefully, we have dedicated a lot of time and major efforts and tried our best to revise the manuscript. We sincerely hope that the revised manuscript can meets your expectations. Once again, thank you very much for your comments and suggestions. Below we have provided a point-by-point response to your valuable comments.

***Comment #1. The abstract is too redundant and more like a research conclusion. Suggest the author to simplify.***

**Response:** Thank you very much for your precious comments. According to your helpful comments, we modified some sentences in the abstract and simplified the abstract, the word count of the abstract before the modification is 324 and after the modification is 214. We hope the revised abstract can meet your satisfaction. The revised abstract are as follows:

**[Pg1, Ln10] to [Pg2, Ln4]: “**The low satisfaction of fans' spectator experience, the weak willingness to continue to watch games have become the realistic barriers limiting the sustained and favorable development of Chinese Basketball Association league (CBA League). Using analysis method SEM and fsQCA, this study clarifies the complex causal relationship behind the phenomenon of fans' spectator experience, explore the linkage mechanism between different influencing factors of spectator experience. Also constructs a variety of grouping paths to enhance the fans' spectator experience in the CBA League. The results shows that Sense, Feel, Think, Act, Relate and Spectator Service all positively affect the satisfaction of the live Spectator experience in the CBA League. Among six influencing factors, none of them is the necessary condition for high satisfaction of Fans’ Spectator Experience. In this way, the characteristics of multi-factors and different paths of the live Spectator experience were verified. Influential factors were coupled to form six grouping paths for high satisfaction of the fans’ spectator experience, which were categorized into two types of configurations by combining the grouping characteristics: 1) Led by spectator service, feel and act experience co-driven, 2) Spectator service and think experience co-driven. The findings provide theoretical guidance for enhancing fan experience and meeting the practical needs of cultivating loyal CBA fan groups while expanding the league's influence.**”**

***Comment #2. There is a lack of relevant literature as a basis in the research background, and the author needs to search for relevant literature as a supplement.***

**Response:** We sincerely appreciate your valuable time and comments and apologize for our carelessness! According to your constructive comments, we added some relevant research literature as a supplement to research background. We hope our solution to this issue can meet with your satisfaction. The revised research background as follows:

**[Pg2, Ln6-22]: “**As the highest-level men's basketball professional league in China, the China Basketball Association League (CBA League) has become a professional league with high influence in China after nearly 30 years of development, and also shoulders the mission and task of reserving and delivering basketball reserve talents for the country [1]. However, the low loyalty of the fan base [2], low consumption desire [3], and low attendance rate of the live games still inhibit the rapid development of the CBA league and the enhancement of its profitability [4], and it has become an urgent problem to improve the experience of the league's fans in the live games in order to increase the number of people who watch the games on-site, thus promoting the rapid growth of the profitability of the CBA league. Whether it is the regular season, playoffs or all-star games, the number of on-site spectators is low, and the profitability of clubs is weak. The reason for this involves a variety of factors, including the level of the game, the operation of the game, the experience of watching the game and brand building [5]. From the perspective of practical implication, enhancing fans' on-site viewing experience lays the foundation for cultivating a loyal fan base, expanding the attractiveness of the league, and stimulating the development of the league's derivative economy [6].**”**

***Comment #3. Provide a detailed description of the research methodology for fsqca,*** [***https://doi.org/10.3390/systems12020053***](https://doi.org/10.3390/systems12020053)***.***

**Response:** We really appreciate for your constructive comments. According to your valuable comment, we provide a detailed description of the fsQCA method in the revised manuscript and cited the recommended research article. The detailed description of fsQCA are as follows:

**[Pg23, Ln1-4]: “**Qualitative comparative analysis (QCA) is based on Boolean operations and set theory as a methodology, which focuses more on the configurational paths formed by multiple combinations of antecedent conditions on the outcome variable than traditional statistical tests that emphasize the “net effect” and have sufficient explanatory power [59].**”**

**Recommended research article:** Dou Z, Sun Y. System Identification of Enterprise Innovation Factor Combinations—A Fuzzy-Set Qualitative Comparative Analysis Method. *Systems*. 2024; 12(2):53.

***Comment #4. Suggest focusing on “Experience Economy Theory and Experiential Marketing” conducts different configuration analyses.***

**Response:** On behalf of my co-authors, we sincerely appreciate your valuable and constructive suggestions! Regarding this comment, we would like to provide some explanations for your reference and hope that our explanation is acceptable to you! Experiential marketing mainly consists of five parts: Feel experience, Sense experience, Think experience, Act experience, and Relate experience. In this study, experiential economic theory and experiential marketing are only used as the theoretical basis to provide support for the construction of the data analysis model. In the subsequent analysis of the group path, combining the experiential economy theory and experiential marketing, we carried out different configurations for the marketing of the CBA league, for example, for different types of fan groups, different marketing strategies should be adopted, strengthening the marketing of sensory experience or strengthening the marketing of emotional experience, and at the same time, improving the construction of the fan service system. In addition, this study constructs a theoretical analysis model based on experiential marketing strategy.
